# Automatized Hepatic Tumor Volume Analysis of Neuroendocrine Liver Metastases by Gd-EOB MRI—A Deep-Learning Model to Support Multidisciplinary Cancer Conference Decision-Making

**DOI:** 10.3390/cancers13112726

**Published:** 2021-05-31

**Authors:** Uli Fehrenbach, Siyi Xin, Alexander Hartenstein, Timo Alexander Auer, Franziska Dräger, Konrad Froböse, Henning Jann, Martina Mogl, Holger Amthauer, Dominik Geisel, Timm Denecke, Bertram Wiedenmann, Tobias Penzkofer

**Affiliations:** 1 Department of Radiology, Charité-Universitätsmedizin Berlin, 13353 Berlin, Germany; alexander.hartenstein@charite.de (A.H.); timo-alexander.auer@charite.de (T.A.A.); franziska.draeger@charite.de (F.D.); konrad.froboese@charite.de (K.F.); dominik.geisel@charite.de (D.G.); tobias.penzkofer@charite.de (T.P.); 2 Division of Gastroenterology, Medical Department, Charité-Universitätsmedizin Berlin, 10117 Berlin, Germany; siyi.xin@charite.de (S.X.); henning.jann@charite.de (H.J.); bertram.wiedenmann@charite.de (B.W.); 3 Bayer AG, 13353 Berlin, Germany; 4 Berlin Institute of Health, 10178 Berlin, Germany; 5 Department of Surgery Campus Charité Mitte/Campus Virchow-Klinikum, Charité-Universitätsmedizin Berlin, 10117 Berlin, Germany; martina.mogl@charite.de; 6 Department of Nuclear Medicine, Charité-Universitätsmedizin Berlin, 13353 Berlin, Germany; holger.amthauer@charite.de; 7 Department of Diagnostic and Interventional Radiology, University Hospital Leipzig, 04103 Leipzig, Germany; Timm.Denecke@medizin.uni-leipzig.de

**Keywords:** neuroendocrine neoplasms, liver metastases, MRI, automatized quantification, deep learning, multidisciplinary cancer conference

## Abstract

**Simple Summary:**

Quantification of liver metastases on imaging is of utmost importance in therapy response assessment, wherein gadoxetic acid (Gd-EOB)-enhanced magnetic resonance imaging (MRI) shows the highest accuracy. Common criteria for assessing therapy response simplify measuring liver metastasis, as full 3D quantification is very time-consuming. Therefore, we trained a deep-learning model using manual 3D segmentation of liver metastases and hepatic parenchyma in 278 Gd-EOB MRI scans of 149 patients with neuroendocrine neoplasms (NEN). The clinical relevance of the model was evaluated in 33 additional consecutive patients with NEN and liver metastases, comparing the model’s segmentation of baseline and follow-up examinations with the therapy response evaluation of an expert multidisciplinary cancer conference (MCC). The model showed high accuracy in quantifying liver metastases and hepatic tumor load, and its measurements matched the response evaluation of an MCC so that its use to support treatment decision-making would be possible.

**Abstract:**

Background: Rapid quantification of liver metastasis for diagnosis and follow-up is an unmet medical need in patients with secondary liver malignancies. We present a 3D-quantification model of neuroendocrine liver metastases (NELM) using gadoxetic-acid (Gd-EOB)-enhanced MRI as a useful tool for multidisciplinary cancer conferences (MCC). Methods: Manual 3D-segmentations of NELM and livers (149 patients in 278 Gd-EOB MRI scans) were used to train a neural network (*U*-Net architecture). Clinical usefulness was evaluated in another 33 patients who were discussed in our MCC and received a Gd-EOB MRI both at baseline and follow-up examination (*n* = 66) over 12 months. Model measurements (NELM volume; hepatic tumor load (HTL)) with corresponding absolute (Δ_abs_NELM; Δ_abs_HTL) and relative changes (Δ_rel_NELM; Δ_rel_HTL) between baseline and follow-up were compared to MCC decisions (therapy success/failure). Results: Internal validation of the model’s accuracy showed a high overlap for NELM and livers (Matthew’s correlation coefficient (φ): 0.76/0.95, respectively) with higher φ in larger NELM volume (φ = 0.80 vs. 0.71; *p* = 0.003). External validation confirmed the high accuracy for NELM (φ = 0.86) and livers (φ = 0.96). MCC decisions were significantly differentiated by all response variables (Δ_abs_NELM; Δ_abs_HTL; Δ_rel_NELM; Δ_rel_HTL) (*p* < 0.001). Δ_rel_NELM and Δ_rel_HTL showed optimal discrimination between therapy success or failure (AUC: 1.000; *p* < 0.001). Conclusion: The model shows high accuracy in 3D-quantification of NELM and HTL in Gd-EOB-MRI. The model’s measurements correlated well with MCC’s evaluation of therapeutic response.

## 1. Introduction

The incidence of neuroendocrine neoplasms (NEN) has increased in the past 30 years considerably, while at the same time, multiple treatment options are available for this disease [1]. The radiological workload for follow-up of patients with NENs has, therefore, increased accordingly. However, not only because of the increasing incidence but also because of the lower aggressiveness of NENs compared to liver metastases of other entities (e.g., colorectal carcinoma), the number of follow-up examinations is increasing [2,3,4,5,6,7,8,9]. Based on the indolent clinical course of NENs, patients often present at an advanced stage for first diagnosis [4,6,9,10]. The liver represents the predominant site for metastases, and accurate calculation of the hepatic metastatic tumor burden is mandatory for therapeutic follow-up [11]. The measurement of diffuse liver lesions, which occur in 60–70% of patients, can be challenging and is–at present-time-consuming. A further challenge is that common therapeutic response criteria intended to characterize how metastases develop over time are not always suitable for each patient [9]. Response criteria in solid tumors (RECIST, Version 1.1) are based on changes in diameters of a few lesions, which are considered representative [12]. However, hepatic tumor load (HTL), which is neglected if only measuring the diameter of metastases, is an important prognostic marker in hepatically metastasized NEN [4,13,14,15,16]. The quantitative evaluation of the metastatic volume can potentially provide a practical method for assessing the disease’s course and may show improved prognostic value.

Magnetic resonance imaging (MRI) is the most sensitive technique to detect and quantify neuroendocrine liver metastases (NELMs) compared to conventional computed tomography (CT), ultrasound (US), and somatostatin receptor imaging [16,17,18]. Gadoxetic acid-enhanced (Gd-EOB) MRI is even more sensitive than conventional extracellular gadolinium chelate-enhanced MRI [17,19,20]. In addition to the use of contrast-enhanced MRI, the use of diffusion-weighted imaging (DWI) sequences increases the sensitivity in the detection of NELM [21,22,23]. Thus, the combination of DWI and hepatobiliary phase (HBP) sequences with Gd-EOB is now the imaging modality with the highest sensitivity for NELM [24]. Hepatic metastases of NELM typically demonstrate a hypervascularization pattern in dynamic contrast phases (arterial, portal-venous and transitional phase), which aids in the differentiation of NELM from other liver lesions [19,25,26]. Despite the value of dynamic contrast phases in differential diagnoses, lesion measurement, and thus response evaluation, is preferably performed in the hepatobiliary phase (HBP) when hepatocyte-specific contrast agents are used [27].

Advances in artificial intelligence (AI) technology have led to generating image recognition algorithms poised to aid and improve medical imaging procedures. AI has already demonstrated strong performance in various medical applications, especially in image-based diagnoses [28]. Although several studies suggest that the performance of AI in imaging diagnosis is superior to human experts, the consensus is that AI should play a supporting role to radiologists and that AI tools could especially be used to save time in clinical routine [28,29,30,31,32,33]. The various fields of AI support in liver imaging include segmentation, lesion detection and classification of diffuse or focal liver diseases [34,35].

Here we provide the first data using a high-precision AI algorithm for the 3D quantification of the hepatic tumor burden of NELM and provide a useful tool for clinical decision-making, for example, in multidisciplinary cancer conferences (MCC).

## 2. Material and Methods

### 2.1. Patient Cohorts

#### 2.1.1. AI Development (AI dev) Cohort

398 MRI scans in 149 patients with NEN, who underwent Gd-EOB enhanced MRI (MAGNETOM Aera (1.5T), Siemens Healthcare, Erlangen, Germany) between January 2015 and August 2018 at our institution were retrospectively identified from our radiology database. 120 of these scans were not suitable for the model’s training because of missing evidence of NELM (*n* = 112) or due to non-standard scan protocols (*n* = 8), resulting in a total inclusion of 278 Gd-EOB MRI datasets. Pretreatments (e.g., partial liver resection, transarterial or local ablative therapies), which may influence the morphology of the liver, were not an exclusion criterion.

#### 2.1.2. MCC Cohort

In a second institutional database search, we consecutively identified 33 patients discussed in our MCC between January 2019 and January 2020 and received a Gd-EOB MRI both as a baseline and as a follow-up examination (*n* = 66). All 33 patients had liver metastases and were selected independently of the hepatic tumor volume or their disease history. In these patients, all MCC decisions were based on the course of the metastatic liver disease. Patients in whom the MCC decision was based on extrahepatic tumor manifestations were excluded.

### 2.2. Magnetic Resonance Imaging

AI dev cohort: MRI was obtained with 1.5 T using phased-array body coils in all patients (MAGNETOM Aera, Siemens Healthcare, Erlangen, Germany) at the same institution. All patients received Gd-EOB (Primovist, Bayer, Berlin, Germany) as an intravenous contrast agent (0.025 mmol/kg body weight; automatic injection at 2 mL/s flow rate, 40 mL saline flush). All MRI examination protocols comprised a 3D T1-weighted (T1w) gradient echo (GRE) sequence with fat saturation (FS) during hepatobiliary contrast phase (HBP) (VIBE: “volumetric interpolated breath-hold examination”; repetition time (TR): 4.58 ms; echo time (TE): 2.21 ms; slice thickness 3 mm, flip angle (FA): 25°; acquisition matrix: 320\0\0\165). The HBP sequence was acquired 20 mins after contrast administration.

MCC cohort: Gd-EOB MRI scans were performed on five different institutional MRI scanners and included both 1.5 T and 3 T examinations. All examinations contained a 3D T1w GRE FS sequence during HBP. Due to the different scanners, the scan parameters (TR, TE, FA and matrix) varied between the examinations. The HBP sequence was acquired between 10 and 20 mins after contrast administration. Among others, diffusion-weighted imaging (DWI) sequences were acquired in the time between Gd-EOB injection and the HBP sequence. All DWI sequences contained at least two b values (b = 0 and b = 800) [36].

All examination protocols corresponded to the ENETS consensus guidelines for the standard of care in neuroendocrine tumors [15].

### 2.3. Manual Segmentation

All HBP sequences of the MRI scans (AI dev and MCC cohort) were anonymized and segmented using the Medical Imaging Interaction Toolkit (MITK) [37]. Volumetry (3D segmentation) of the liver and all liver metastases was performed in the HBP 3D T1w-GRE FS sequence. There was no limit on the number of metastases segmented per patient. Segmentation was performed manually using the polygonal region of interest (ROI) tool and is based on the planimetry method. Margins of the liver metastases were defined by the signal difference between hypointense liver metastases and the contrast-enhanced liver parenchyma. Adjacent vessels and biliary ducts were excluded if reasonably possible. All segmentations were refined by a radiologist with >5 years of experience in abdominal MRI. Distribution patterns of NELM were scored according to the number: singular, multiple (≤10 metastases) and diffuse (>10 metastases) and distribution: unilobar (left or right) and bilobar.

For subanalysis, all NELM and livers were manually segmented in DWI sequences in the MCC cohort. The segmentation process was equivalent to that previously described in the HBP sequences.

### 2.4. Model Training and Validation

The model was trained using the MIC-DKFZ nnU-Net (Division of Medical Image Computing, German Cancer Research Center (DKFZ), Heidelberg, Germany) deep-learning framework. nnU-Net is an open-source tool. The source code and comprehensive documentation are publicly available on GitHub [38]. nnU-Net enables 3D semantic segmentation in many biomedical imaging applications without requiring designing respective specialized solutions [39]. Out of the 278 MRI scans, 222 (80%) HBP sequences were randomly chosen for the model training.

The HBP sequences of the remaining 56 scans (20%) were used to test the model’s accuracy (internal validation). External validation of the model’s accuracy was performed in the MCC cohort (different scanners (1.5 T and 3 T) and sequence parameters were used compared to the model’s training).

### 2.5. Clinical Correlation

Our model analyzed the NELM volume and liver volume of the 33 patients with MCC decisions in the baseline (BL) scan and the follow-up (FU) scan on which the MCC decisions were based. The MCC is part of our European Neuroendocrine Tumor Society (ENETS) center of excellence and consists of specialized gastroenterologists, endocrine surgeons, pathologists, nuclear medicine specialists, radiotherapists and radiologists. Absolute and relative changes in NELM volume and HTL calculated by the model were analyzed and compared to the MCC decisions. MCC decisions were classified as therapy success (stable disease (SD) or partial regression (PR)) or therapy failure (progressive disease (PD)) based on the presented images. The evaluation within the board was guided by the response criteria in solid tumors (RECIST, Version 1.1).

### 2.6. Statistics

Statistical analysis was performed using SPSS Statistics (IBM, Version 25, Armonk, NY, USA). The Kolmogorov–Smirnov test showed a non-normal distribution of the data. Therefore, nonparametric testing was performed.

Descriptive data were accordingly presented as the median and interquartile range (IQR). Relative size differences in segmentations were calculated by the following formula: (model’s volume–radiologists’ volume)/radiologists’ volume. Matthew’s correlation coefficients (φ) were calculated to measure the model’s segmentation accuracy as previously published [40]. MCC decisions were compared to the automatized volume evaluation of the model. HTL was calculated by the formula: (NELM volume/(liver volume–NELM volume)) × 100. Absolute volume changes were calculated by the difference: Volume_Follow-up_–Volume_Baseline_. Relative volume changes were calculated by the formula: ((Volume_Follow-up_–Volume_Baseline_)/Volume_Baseline_) × 100. Mann–Whitney *U* test was used as a dominance test comparing two independent groups of quantitative data. A sign test was used to compare two related samples. Spearman’s rank test was used for correlation analysis in continuous variables, and the corresponding correlation coefficients (r_s_) were calculated. ROC analysis was performed, and Youden indexes were calculated to determine optimal cutoff values.

## 3. Results

### 3.1. Patient Cohorts

#### 3.1.1. AI dev Cohort

Characteristics of the 149 patients with NEN are summarized in Table 1. The most common primary tumor sites were the ileum (51.0%) and the pancreas (43.0%). Confirmed (histologically or with the aid of SR imaging) liver metastases were present in 118 patients (79.2%), which were used for the model training. Out of these 118 patients, 4 patients (3.4%) had singular liver metastasis, 59 patients (50%) had multiple metastases (≤10 metastases), and 55 patients (46.6%) had a diffuse metastatic pattern (>10 metastases). Both liver lobes were involved in 91 patients (77.1%). Unilobar disease limited to a single liver lobe was found in 27 patients (22.9%) (right liver: 24 patients, left liver: 3 patients).

#### 3.1.2. MCC Cohort

Characteristics of the 33 patients with MCC decisions are summarized in Table 1. Comparably to the training cohort, the most common primary tumor sites were the pancreas and ileum (36.4% each). Therapeutic response was classified by the MCC as therapeutic success in 16 (48%) patients (SD: *n* = 14; PR: *n* = 2) and therapeutic failure in 17 (PD, 52%) patients.

### 3.2. Validation of the Model

**Figure 1:**
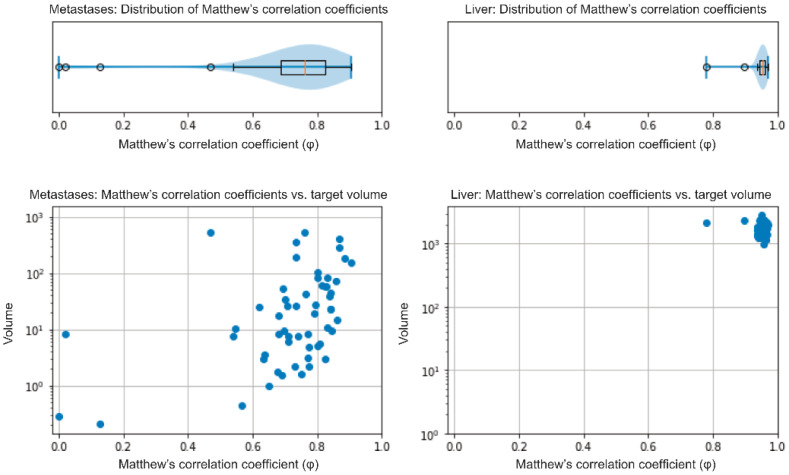
Internal validation–distribution of φ in NELM and liver segmentations (upper row) and its distribution in correlation to the target volume in cm^3^ (lower row).

The median NELM volume in the 56 patients (internal validation) of the AI dev group was 17.25 cm^3^ (IQR: 4.48–60.93 cm^3^) as determined by the nnU-Net model and 16.17 cm^3^ (IQR: 4.87–58.16 cm^3^) in the radiologists’ manual segmentation. The median relative volume difference between the model’s and the radiologist’s segmentation of NELM was −3.7% (IQR: −24.54−+11.83%). The model showed a median φ of 0.76 (IQR: 0.68–0.83) for the segmentation of metastases (Figure 1, left side). Analysis of the data in a case-wise fashion identified three out of 56 patients (5.4%), whereby the model’s segmentation only achieved a weak overlap (φ < 0.2). Two out of these patients had very low NELM volume (0.1 and 0.2 cm^3^). The third patient showed atypical, hyperintense signal intensities of the metastases in HBP; NELM were subsequently missed by the model. Dividing the cohort by the median NELM volume (16.17 cm^3^) into high and low NELM volume, the model showed significant higher φin patients with higher NELM volume (median φ: 0.80; IQR: 0.73–0.84) compared to low NELM volume (median φ: 0.71; IQR: 0.64–0.78; *p* = 0.003). For liver segmentation, the median volume was 1639.9 cm^3^ (IQR: 1366.1–1960.7 cm^3^) in the manual segmentation and 1659.0 cm^3^ (IQR: 1404.2–1966.9 cm^3^) in the model’s segmentation. The median relative volume difference between the model’s and the manual segmentations of livers was +0.9% (IQR: −0.7−+4.2%). The model showed a median φ of 0.95 (IQR: 0.95–0.96) in liver segmentation (Figure 1, right side). The external validation (MCC cohort) confirmed the high accuracy of the model. The model achieved a median φof 0.86 (IQR: 0.81–0.91) in the segmentation of NELM and of 0.96 (IQR: 0.95–0.96) in liver segmentation.

### 3.3. Automatized NELM Volume Analysis and Clinical Correlation (MCC Cohort)

**Figure 2:**
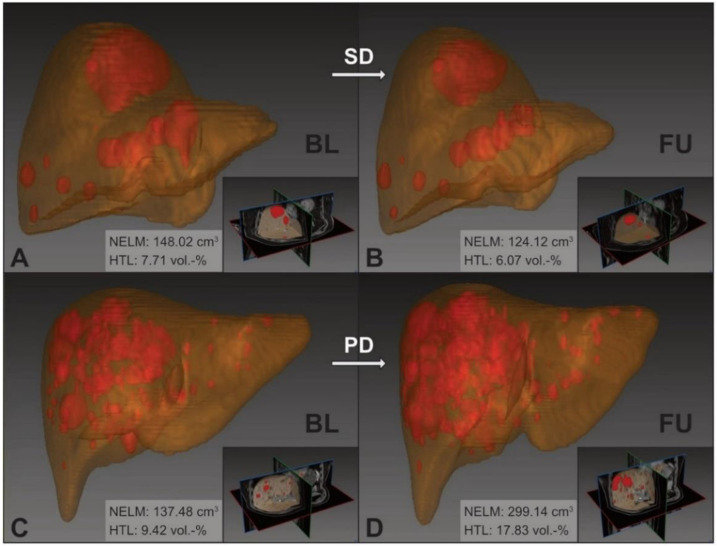
nnU-Net 3D segmentation of NELM and livers in the MCC cohort. Upper row: example images of therapy success (patient ID: 11) with stable disease between baseline (**A**) and follow-up (**B**); Δ_rel_NELM: −16.14% and Δ_rel_HTL: −21.23%. Lower row: example images of therapy failure (patient ID: 08) with progressive disease between baseline (**C**) and follow-up (**D**); Δ_rel_NELM: +117.58% and Δ_rel_HTL: +89.32%. BL: baseline; FU: follow-up; SD: stable disease; PD: progressive disease; NELM: neuroendocrine liver metastasis; HTL: hepatic tumor load.

The model’s measurements of the MCC cohort are summarized in Table 2 and exemplarily visualized in Figure 2.

**Figure 3:**
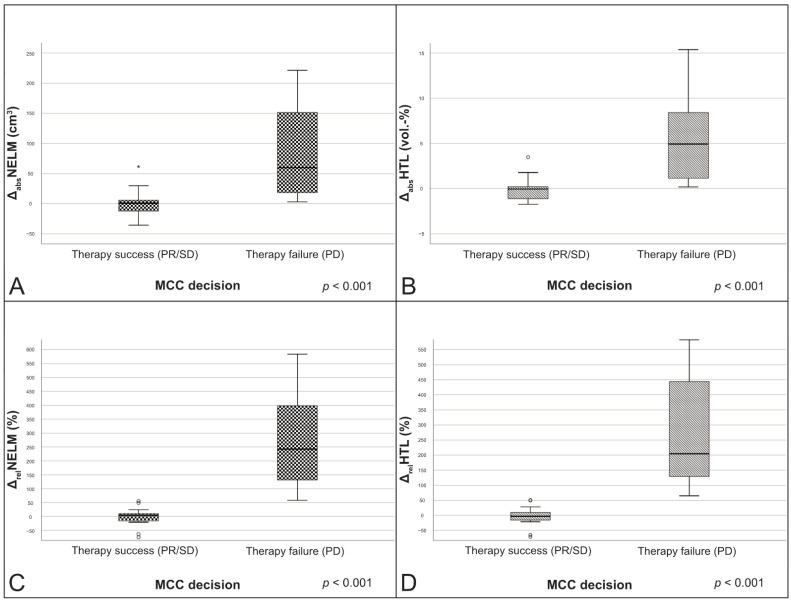
Boxplots of the change variables in correlation to the MCC decisions. (**A**) Δ_abs_NELM; (**B**) Δ_abs_HTL; (**C**) Δ_rel_NELM; (**D**) Δ_rel_HTL. PR: partial response; Δ_abs_NELM: absolute NELM volume change; Δ_abs_HTL: absolute HTL change; Δ_rel_NELM: relative NELM volume change; Δ_rel_HTL: relative HTL change.

The comparison between patients with therapy success (*n* = 16) and therapy failure (*n* = 17) showed significant differences for all absolute and relative volume changes (*p* < 0.001). Patients classified as therapy success by the MCC showed significant lower values in median absolute NELM volume change (Δ_abs_NELM), median absolute HTL change (Δ_abs_HTL), median relative NELM volume change (Δ_rel_NELM) and median relative HTL change (Δ_rel_HTL) than patients with therapy failure (*p* < 0.001) (Figure 3).

**Figure 4:**
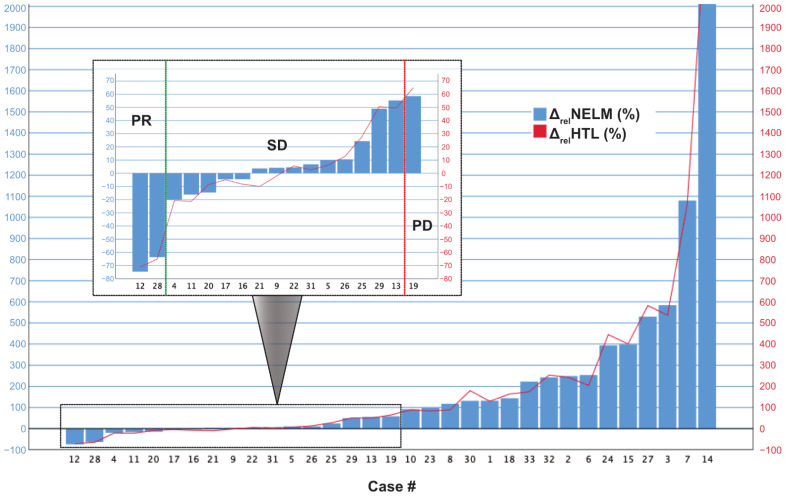
Case-wise illustration of the relative volume changes (Δ_rel_NELM and Δ_rel_HTL) between baseline and follow-up examination in the MCC cohort. The box within the figure shows an optimized scaling of cases with less change to illustrate the significance thresholds for partial response (PR) and progressive disease (PD).

The case-wise analysis of the 33 MCC patients is summarized in Table 3. The case-wise analysis showed that the model correctly detected increased NELM volume in all of the 17 patients with therapeutic failure (100%). The Δ_abs_NELM increase in these 17 patients ranged from +3.02 cm^3^ to +864.45 cm^3^ and Δ_abs_HTL ranging from +0.18 vol.-% to +36.41 vol.-%. The relative increase of Δ_rel_NELM ranged from +58.52% to +4513.64% and in Δ_rel_HTL from +64.97% to +2497.20%. In patients with therapeutic success (*n* = 16), the Δ_abs_NELM ranged from −394.57 cm^3^ to −34.75 cm^3^ (in PR) and −35.70 cm^3^ to +61.56 cm^3^ (in SD) and the Δ_abs_HTL from −16.96 vol.-% to −1.73 vol.-% (in PR) and −1.64 vol.-% to +3.48 vol.-% (in SD). The relative change variables of Δ_rel_NELM ranged from −74.68% to −63.68% in PR and from −20.19% to 55.25% in SD, and the Δ_rel_HTL ranged from −71.13% to −65.03% in PR and from −21.23% to +50.51% in SD (Figure 4).

**Figure 5:**
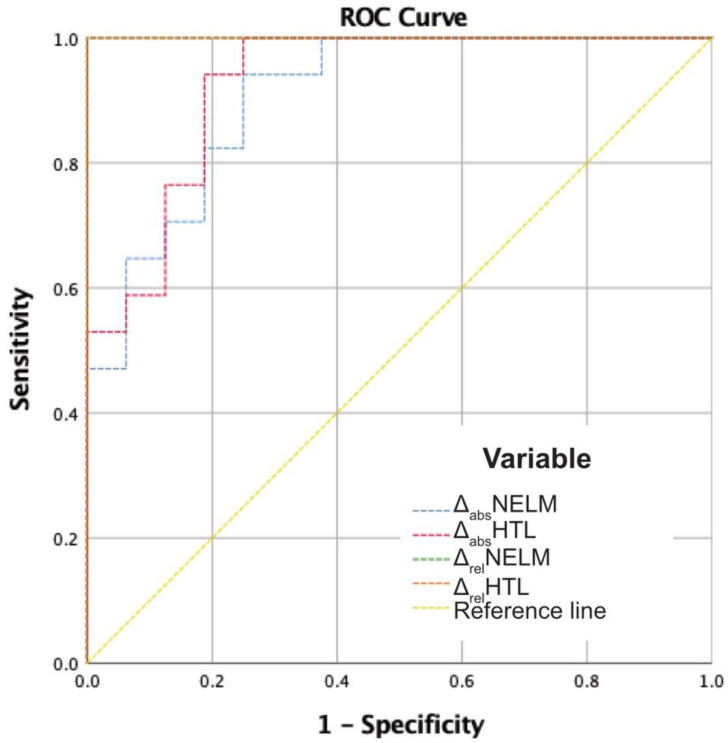
ROC analysis of the absolute and relative change variables in relation to the MCC decisions. AUC Δ_abs_NELM: 0.908; AUC Δ_abs_HTL: 0.926; AUC Δ_rel_NELM: 1.000; AUC Δ_rel_HTL: 1.000; *p* < 0.001.

In the total cohort, ROC analysis of MCC decision and the relative changes (Δ_rel_NELM and Δ_rel_HTL) showed an area under the curve (AUC) of 1.000 (*p* < 0.001) for both variables. The absolute changes showed an AUC of 0.908 for Δ_abs_NELM and of 0.926 for Δ_abs_HTL (*p* < 0.001). To determine the best cutoff values for progressive disease, a Youden index was calculated. For Δ_rel_NELM, the highest Youden index (1.000; 100% sensitivity and 100% specificity) was found at the cutoff value +56.88%. For Δ_rel_HTL, the highest Youden index (1.000; 100% sensitivity and 100% specificity) was found at a cutoff value of +57.73% (Figure 5).

### 3.4. Comparison of 3D Quantification between HBP and DWI Sequences

In the MCC cohort, manual segmentations of NELM (r_s_: 0.981; *p* < 0.001), livers (r_s_: 0.966; *p* < 0.001) and HTL (r_s_: 0.956, *p* < 0.001) showed a high correlation between HBP and DWI sequences. However, direct comparison of the measured values for NELM and livers showed significant differences between HBP and DWI (*p* < 0.001; Table 4). When looking at the changes between BL and FU, a high correlation between DWI and HBP sequences was also shown for Δ_abs_NELM (*r_s_*: 0.919; *p* < 0.001), Δ_rel_NELM (*r_s_*: 0.960; *p* < 0.001), Δ_abs_HTL (*r_s_*: 0.883, *p* < 0.001) and Δ_rel_HTL (*r_s_*: 0.952; *p* < 0.001). There were no significant difference of Δ_abs_NELM, Δ_rel_NELM, Δ_abs_HTL and Δ_rel_HTL between DWI and HBP-based measurements (*p* = 0.072 to 0.719; Table 4).

## 4. Discussion

This is the most extensive study presenting AI data quantifying the total volume of hepatic tumor burden in NEN using a deep-learning model combined with Gd-EOB MRI. The model achieved high accuracy, especially in patients with higher NELM volume and delivered results corresponding to the MCC consensus decision-making regarding therapeutic success or failure.

The presented deep-learning model differs from previous studies in several aspects. First, the training data set of 278 Gd-EOB MRI examinations is the largest published to date in the automated assessment of focal liver lesions [41]. The high proportion of patients with more than ten metastases resulted in more than 2000 segmented metastases. Second, various hepatic conditions were included in the model’s training. Previous liver resection, excessive pretreatment, ablation therapies or preceding intraarterial treatments (e.g., transarterial chemoembolization (TACE) or selective internal radiation therapy (SIRT)) were no exclusion criteria for training. The combination of high case numbers and various pretreatments should improve the robustness of the model in preparation for everyday clinical usage [42,43]. Due to the broad training, it is possible to quantify patients under different therapies with the model. However, individual pitfalls must be considered. Therapy-induced hemorrhage of NELM affects the visualization of lesions in HBP sequences. Our study identified one case in which the model had achieved low accuracy for this reason. In addition, in two cases with very low tumor burden, our model showed only unsatisfactory accuracy. Though, these cases are also of less interest for an automated volume analysis since a conventional, manual evaluation could easily be performed. The aim of our study was not to replace manual evaluation but to complement and improve it.

Our results demonstrate that accurate, automated 3D segmentation of NELM is feasible in HBP from Gd-EOB MRI examinations. Due to the comparatively lower growth dynamics of NELM compared to metastases from other primary tumors, we believe automated quantification is particularly valuable based on the numerous follow-up studies in patients with NEN. Even if NELM is characterized by marked arterial hypervascularization or cystic components on imaging, these features do not affect the HBP sequence [25]. Liver metastases from a wide variety of primary tumors show the same typical imaging characteristics in the HBP sequences with marked hypointensity of the lesion compared to the surrounding liver parenchyma [44]. Therefore, by using HBP in Gd-EOB MRI, our model is not limited to the segmentation of NELM, and its use should also be investigated for liver metastases of other primary tumors.

The high value of Gd-EOB HBP sequences in the determination of NELM size has already been shown and corroborates our approach to using this sequence for 3D segmentation [45]. The high lesion to liver contrast also provides optimum conditions for automated segmentation [46,47]. However, besides its excellent imaging characteristics, Gd-EOB MRI has some disadvantages. These include the comparatively high costs due to the contrast agent itself and the resulting examination time, the general side effects and possible deposition of gadolinium [48]. As a non-contrast alternative with high sensitivity, DWI sequences can also be effectively used to measure NELM without the disadvantages of Gd-EOB MRI [49,50]. Currently, however, DWI sequences are used for detection rather than measurements of liver lesions. In particular, a 3D measurement may be limited by the lower axial resolution of commonly used DWI sequences. In our subanalysis, we could show that DWI-based measurements correlate strongly with those in HBP. However, the absolute measurements of NELM and livers showed significant differences between the two sequences, so that an exact 3D quantification using DWI was not possible. Nevertheless, this inaccuracy was relativized when the measurements were compared in evaluating treatment response. The relative and absolute changes of NELM volume and HTL between baseline and follow-up examination showed no significant difference between HBP and DWI so that evaluation of treatment response using 3D measurements in DWI seems feasible. Therefore, the results of our study encourage developing similar automation for non-contrast DWI MRI as well.

The limitation to lesion diameters versus volume in clinical routine can be best explained by the time required for full 3D volumetry. Up to now, 3D volumetry of liver lesions has only been carried out within the framework of studies [51,52]. Besides the volumetric assessment of tumor burden, the 3D segmentations generated by the model presented in this study could be used for further lesion analysis, such as texture analysis, radiomics or contrast-uptake used in Choi criteria [53,54]. To date, most studies concerning artificial intelligence and liver imaging focus on diffuse liver disease or the classification of liver tumors [55,56,57]. With the help of the presented model and the associated time saving by the automatized segmentation, not only 3D quantification of HTL but also more sophisticated tumor analyses could find their way into clinical routine.

Assessment of therapeutic response in liver metastases, independently from primary tumor origin, is most commonly based on the Response Evaluation Criteria in Solid Tumors (RECIST, Version 1.1). RECIST1.1 is suitable for study cohorts and facilitates response evaluation by defining a limited number (maximum two per organ) of target lesions [58]. From a practical point, response criteria vary regarding increasing versus decreasing tumors. Partial response (PR) is defined as a decrease of at least 30% in the sum of the largest diameter of target lesions. By contrast, progressive disease is defined as increasing at least 20% of target lesions or the appearance of one or more new lesions in a 2D measurement [59]. Considering this somehow simplified approach, the pure volumetric determination of growth behavior should allow a more precise measuring method for therapeutic decision-making in the individual patient. The simplification of RECIST1.1 can lead to patients being interpreted incorrectly or inconsistently during their illness. The limitations of RECIST1.1 become even more evident when evaluating the effects of targeted molecular agents, especially in slow-growing tumors, such as NENs [60,61]. RECIST1.1 treatment response strongly depends on which target lesions were chosen at the baseline scan. Heterogeneous treatment response, which can be seen in different types of primary cancers and systemic treatments, is not represented by RECIST1.1 [62]. Additionally, volumetric measurement methods show a higher intra-observer reproducibility compared to RECIST1.1 [63]. Quantification of total HTL in clinical routine is not routinely performed, and in most cases, tumor load is visually estimated by the radiologist. However, several studies have shown that hepatic tumor burden is an important prognostic imaging marker [13,64,65]. Volumetric evaluation of the HTL, as performed by our model, provides useful information on lesion distribution and allows a more realistic quantification of hepatic tumor extent than the (2D) diameter measurements, which are commonly used [66]. In addition, the model considers all lesions, which would also allow capturing of heterogeneous treatment responses.

The new challenge in volumetric tumor mass determination will be developing new cutoff values. If metastasis is seen as a sphere mathematically, an increase of the diameter of the lesion of 20%, which defines a lesion to be classified as a progressive disease in RECIST1.1, would result in a volume increase of approximately 73%. In our cohort, the MCC stated progressive disease and therapy failure when the tumor volume, as determined by the model, increased by 57%. Furthermore, a NELM volume decrease of −57% correctly identified the two patients with partial response. Our results show that 3D assessment of NELM could be useful, but further studies are needed to evaluate its superiority over 2D methods regarding clinical endpoints [67].

MCCs are designed to optimize patient outcomes by elaborating the best treatment plans or changes in cases of therapy failure in a multidisciplinary approach [68,69]. The number of cases discussed in each MCC is steadily rising. This can be explained by the increasing acceptance of the multidisciplinary approach and the rising incidence of cancers due to improved diagnostics [70]. Our study shows that deep-learning models can assist the MCC’s decisions by automatized the quantification of HTL. Besides the time-saving aspect, the model could also provide decision support to physicians who have no access to a regularly held MCC.

Our study has some limitations. As mentioned above, the 3D assessment approach needs to be further evaluated on larger clinical collectives with direct comparison to 2D measurements and the impact on clinical endpoints. Another limitation of the study is that the ground truth of accuracy is based on manual segmentation of liver metastasis. Due to the sometimes pronounced, even small foci of liver metastases, manual segmentation is not perfect. To minimize this limitation, all segmentations were checked multiple times to capture all metastases (no limit on the number of lesions per patient) and to train the model as realistically as possible.

## 5. Conclusions

In conclusion, the deep-learning model presented shows high accuracy in 3D volumetry of NELM and determination of HTL in Gd-EOB MRI and paves the way for fully automated 3D assessment of hepatic disease. The model also provides useful (potentially prognostic) information about HTL and NELM volume and can be used to assist physicians in response evaluation and the decision-making about therapeutic success or failure comparable to the decisions of an expert multidisciplinary cancer conference.

## Figures and Tables

**Table 1:** Patient and disease characteristics of the AI dev cohort and MCC cohort.

Feature	Subgroups	AI dev Cohort	MCC Cohort	*p*-Value
Number of patients	-	149	33	-
Number of scans	-	278 (of 398)	66	-
Gender (M: F)	-	66:83	18:15	0.285
Age (median)	-	58.92 (48.86–66.38)	56.45 (48.62–67.40)	0.631
Ki67 (%, median)	-	5.0 (2.0–10.0)	7.0 (2.5–13.0)	0.139
Primary site				0.001
	Pancreas Ileum Other	64 (43.0%) 76 (51.0%) 9 (6.0%)	12 (36.4%) 12 (36.4%) 9 (27.2%)	
Grading				0.406
	1 2 3	52 (34.9%) 85 (57.0%) 12 (8.1%)	8 (24.2%) 23 (69.7%) 2 (6.1%)	
NET: NEC	-	144:5	31:2	0.612
Functionality	yes no	42 (28.2%) 107 (71.8%)	12 (36.4%) 21 (63.6%)	0.401
Extrahepatic metastases	-	92 (61.7%)	27 (81.8%)	0.042
Somatostatin receptor (SR)				0.004
	pos neg	110 (73.8%) * 37 (24.9%) *	32 (97.0%) 1 (3.0%)	

* no SR imaging available in 2 patients (1.3%), Data were presented as *n* (%) or median (IQR). *p*-values are based on *χ*^2^ test, Fisher’s exact test or Mann–Whitney *U*-test. AI: artificial intelligence; MCC: multidisciplinary cancer conference; NET: neuroendocrine tumor; NEC: neuroendocrine carcinoma.

**Table 2:** Summary of the model’s segmentation results for the MCC cohort and their absolute and relative changes between baseline and follow-up MRI examinations.

Variable	Overall				Significance
	BL		FU		
*n*	33		33		-
NELM (cm^3^)	23.48 (10.45–113.17)		86.93 (12.08–204.50)		-
Liver (cm^3^)	1582.23 (1336.25–2030.03)		1716.75 (1477.12–2092.94)		-
HTL (vol.-%)	1.57 (0.55–7.05)		5.93 (0.99–11.74)		-
Δ_abs_NELM (%)	14.70 (0.76–96.35)				-
Δ_abs_HTL (%)	0.98 (−0.03–5.41)				-
Δ_rel_NELM (%)	58.51 (3.93–245.64)				-
Δ_rel_HTL (%)	64.97 (−3.44–223.31)				-
	Therapy Success		Therapy Failure		
	BL	FU	BL	FU	
*n*	16	16	17	17	-
NELM (cm^3^)	75.45 (12.35–141.65)	66.78 (11.64–167.82)	19.15 (7.04–78.44)	86.93 (24.40–253.32)	-
Liver (cm^3^)	1692.26 (1475.09–2061.63)	1725.30 (1471.78–2130.28)	1580.35 (1290.13–1902.53)	1716.75 (1451.10–2106.81)	-
HTL (vol.-%)	4.41 (0.87–7.83)	3.75 (0.75–8.88)	1.46 (0.34–5.97)	5.93 (1.47–16.78)	-
Δ_abs_NELM (%)	0.76 (−18.07–39.32)		59.70 (16.49–156.59)		*p* < 0.001
Δ_abs_HTL (%)	−0.03 (−1.28–0.23)		4.94 (1.07–9.78)		*p* < 0.001
Δ_rel_NELM (%)	3.93 (−15.75–10.36)		242.68 (124.56–463.87)		*p* < 0.001
Δ_rel_HTL (%)	−3.45 (−18.11–11.15)		204.49 (109.39–490.19)		*p* < 0.001

Values are displayed as median and interquartile range. *p*-values are based on Mann–Whitney *U*-test. BL: baseline; FU: follow-up; NELM: neuroendocrine liver metastasis; HTL: hepatic tumor load; Δ_abs_NELM: absolute NELM volume change; Δ_abs_HTL: absolute HTL change; Δ_rel_NELM: relative NELM volume change; Δ_rel_HTL: relative HTL change.

**Table 3:** Case-wise summary of the model’s measurements and response variables in the MCC cohort.

Case #	Baseline			Follow-up			Response Variables				
ID	Liver Volume (cm^3^)	NELM Volume (cm^3^)	HTL (vol.-%)	Liver Volume (cm^3^)	NELM Volume (cm^3^)	HTL (vol.-%)	MCC	Δ_abs_NELM (cm^3^)	Δ_abs_HTL (vol.-%)	Δ_rel_NELM (%)	Δ_rel_HTL (%)
0001	1649.3	100.5	6.5	1796.0	232.7	14.9	PD	132.2	8.4	131.6	129.5
0002	1582.2	69.5	4.6	1783.7	242.4	15.7	PD	172.9	11.1	248.6	242.1
0003	1516.3	23.5	1.6	1766.8	160.7	10.0	PD	137.2	8.4	584.3	536.1
0004	1476.7	2.5	0.2	1487.8	2.0	0.1	SD	−0.5	−0.0	−20.2	−20.8
0005	1304.2	9.5	0.7	1350.2	10.5	0.8	SD	0.9	0.1	9.8	6.1
0006	1247.6	87.3	7.5	1656.0	308.8	22.9	PD	221.5	15.4	253.5	204.5
0007	1092.4	0.6	0.1	1111.6	7.0	0.6	PD	6.4	0.6	1080.0	1066.3
0008	1597.5	137.5	9.4	1977.1	299.1	17.8	PD	161.7	8.4	117.6	89.3
0009	1474.5	21.2	1.5	1567.0	22.1	1.4	SD	0.9	−0.0	4.2	−2.0
0010	1579.1	3.3	0.2	1610.6	6.3	0.4	PD	3.0	0.2	91.9	88.5
0011	2067.7	148.0	7.7	2167.6	124.1	6.1	SD	−23.9	−1.6	−16.1	−21.2
0012	1959.7	46.5	2.4	1689.6	11.8	0.7	PR	−34.8	−1.7	−74.7	−71.1
0013	1695.2	111.4	7.0	1817.3	173.0	10.5	SD	61.6	3.5	55.3	49.5
0014	1332.6	19.2	1.5	3216.9	883.6	37.9	PD	864.5	36.4	4513.6	2497.2
0015	2209.1	9.4	0.4	2236.5	47.0	2.2	PD	37.6	1.7	398.2	400.5
0016	2326.4	13.0	0.6	2421.6	12.4	0.5	SD	−0.6	−0.1	−4.5	−8.3
0017	969.3	12.1	1.3	972.4	11.6	1.2	SD	−0.6	−0.1	−4.5	−4.9
0018	2523.2	17.7	0.7	2364.0	43.3	1.9	PD	25.5	1.2	144.2	163.6
0019	1580.4	54.8	3.6	1552.8	86.9	5.9	PD	32.1	2.3	58.5	65.0
0020	1703.4	244.9	16.8	1572.5	209.2	15.3	SD	−35.7	−1.5	−14.9	−8.6
0021	1575.0	114.9	7.9	1801.2	119.2	7.1	SD	4.2	−0.8	3.7	−10.0
0022	1082.3	14.0	1.3	1071.6	14.6	1.4	SD	0.6	0.1	4.5	5.7
0023	2016.6	133.1	7.1	2303.4	264.3	13.0	PD	131.1	5.9	98.5	83.4
0024	1788.5	4.7	0.3	1639.5	22.9	1.4	PD	18.3	1.2	393.3	444.3
0025	2043.5	122.5	6.4	2018.2	152.3	8.2	SD	29.8	1.8	24.3	28.0
0026	2416.2	180.8	8.1	2389.9	199.9	9.1	SD	19.1	1.0	10.5	12.8
0027	1339.9	11.3	0.9	1297.2	71.0	5.8	PD	59.7	4.9	529.6	582.1
0028	2653.8	549.0	26.1	2386.0	199.4	9.1	PR	−349.6	−17.0	−63.7	−65.0
0029	1477.6	7.2	0.5	1466.5	10.8	0.7	SD	3.5	0.3	49.0	50.5
0030	2054.2	11.2	0.6	1716.8	25.9	1.5	PD	14.7	1.0	131.5	179.8
0031	1689.3	104.4	6.6	1761.0	111.5	6.8	SD	7.1	0.2	6.8	2.6
0032	1207.2	62.4	5.5	1322.4	214.0	19.3	PD	151.5	13.9	242.7	253.9
0033	1146.0	2.1	0.2	1349.4	6.7	0.5	PD	4.6	0.3	222.3	174.6

**Table 4:** Comparison of 3D quantification between HBP and DWI sequences.

Variable	HBP	DWI	Significance
NELM volume (cm^3^)	63.24 (12.12–174.23)	76.28 (12.61–182.48)	*p* = 0.002
Liver volume (cm^3^)	1659.28 (1387.73–2052.00)	1595.00 (1324.17–1977.54)	*p* < 0.001
HTL (vol %)	4.05 (0.76–9.23)	5.45 (0.88–11.49)	*p* < 0.001
Δ_abs_NELM (cm^3^)	19.57 (17.27–132.52)	30.06 (18.91–142.13)	*p* = 0.072
Δ_rel_NELM (%)	107.76 (5.28–245.04)	78.35 (11.22–221.21)	*p* = 0.719
Δ_abs_HTL (vol %)	1.20 (−0.01–8.87)	1.25 (0.10–10.47)	*p* = 0.151
Δ_rel_HTL (%)	111.36 (−0.36–254.49)	67.76 (4.20–198.88)	*p* = 0.151

Values are displayed as median and interquartile range. *p*-values are based on the sign test. HBP: hepatobiliary phase; DWI: diffusion weighted imaging; NELM: neuroendocrine liver metastasis; HTL: hepatic tumor load; Δ_abs_NELM: absolute NELM volume change; Δ_abs_HTL: absolute HTL change; Δ_rel_NELM: relative NELM volume change; Δ_rel_HTL: relative HTL change.

## Data Availability

Data is contained within the article.
